# Diagnosis: Fundamental Principles and Methods

**DOI:** 10.7759/cureus.28730

**Published:** 2022-09-03

**Authors:** Martin S Gale

**Affiliations:** 1 Endodontics, Ivanhoe Specialist Endodontics, Melbourne, AUS

**Keywords:** clinical decision rules, decision support techniques, clinical decision making, diagnostic error, diagnostic systems, diagnostic technique

## Abstract

Problem-solving is an essential human endeavor. It involves problem definition, followed by solution design, solution implementation, and, finally, review. Healthcare is a prime and often successful example of problem-solving. In medical terminology, the problem definition is termed the diagnosis, the solution design is called the treatment plan, and the solution itself is the provision of treatment. The case review is used to assess the efficacy of the solution. This process is sequential and consequential. Obviously, if a problem’s definition is absent or incorrect, then an appropriate solution cannot be designed or implemented. Unfortunately, missed or incorrect diagnoses are not uncommon in healthcare and can cause considerable harm and economic cost. Minimizing these diagnostic errors would confer great benefits to patients, clinicians, and healthcare organizations. Understanding the nature of diagnosis and why it can be so difficult are some of the first steps in reducing these diagnostic errors. One issue is that diagnosis may seem to be more of an art than a science when the exact mechanisms of diagnosis are often not clearly described. Clinicians typically learn their diagnostic craft by mimicry of their mentors over years of hard experience. This absence of a clear method not only makes diagnosis difficult to do well it also makes diagnosis difficult to teach well. The purpose of this paper is to try to better articulate the fundamental principles and methods of diagnosis and to make diagnosis more intellectually understandable, and so more teachable, and more achievable by clinicians.

## Introduction and background

Problem-solving is an important part of human activity. Typically, problems are identified, solutions are designed and implemented, and the outcome is reviewed. In healthcare, the main problem to be solved is a disease. Identification of disease is termed diagnosis, the solution design is called treatment planning, and treatment where appropriate is then implemented as the solution. Case review determines the outcome. These four stages are linearly dependent. Therefore, an error in diagnosis can lead to consequent errors in treatment planning, hence in treatment given, which then leads to a poor outcome on review. Incorrect diagnoses may cause patient harm, operator stress, and economic loss [1]. Unfortunately, diagnostic errors are commonplace [2-10]. There is a need to minimize these diagnostic errors and their harm [1-3,9]. This is often difficult when healthcare domains are usually complex and incompletely understood, even by domain experts [2,7,11]. Moreover, effective teaching of diagnostic skills requires that the principles are consciously understood [12]. The aim of this paper is to describe the fundamentals of the diagnostic process to aid its understanding, teaching, and implementation.

## Review

The language of diagnosis

**Figure 1:**
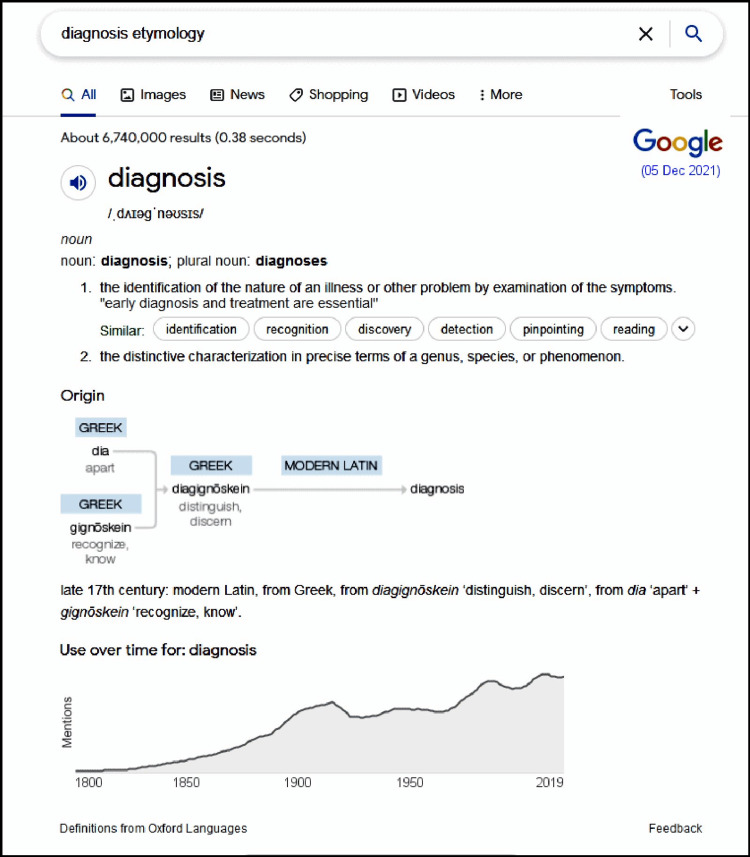
Google search: etymology of the word diagnosis.

The term diagnosis is part of the fabric of medical language, and indeed elsewhere. Despite this ubiquity, many users may be oblivious to the origin of the word, which remarkably tells us its fundamental meaning. It is no accident that in the 1600s the word diagnosis was derived from two Latin words, which, in turn, came from ancient Greek. The English-language versions of the two words are *gnosis* (“to know”) and *dia* (“apart”) (Figure 1) [13]. These two words indicate an ability to recognize (know) one condition as separate (apart) from another; that is, to discriminate between health and disease and between different diseases. That the original literal meaning has endured so precisely into modern times demonstrates the word’s utility.

So, we now know the meaning of the word diagnosis. But the word alone does not instruct us how to make a diagnosis, or how to teach a diagnostic method. Making a diagnosis is done by system analysis.

System analysis

To understand a diagnostic method, it is essential to consider the fundamental system in which a diagnosis is to be made [14]. This can be complicated, so bear with me. At a basic level, our patient is made up of many aggregated complex parts and processes [15]. This complex whole system is difficult to understand when the different parts and processes obscure each other, often confounding the interpretation of cause and effect. The scientific method uses deconstruction to break up that complexity into smaller isolated parts, which can be more easily understood with logic [16]. Once properly understood, these smaller structures and mechanisms are then used to explain the behavior of the full working system as a model. So, to understand the system one has to identify its parts. Those parts can be described as elements or components [14]. These system elements are defined by us as they occur in nature, or at least as we perceive them.

Each element in the system can be working well or may be malfunctioning. In a healthy system, all elements will be in their correct state. Alternatively, if one or more elements are malfunctioning, then the system may malfunction, and in the medical sense, the disease is present [14]. Many of these elements may be unrelated, but others may be linked, such that when one element varies then another element changes as well. Such a linkage between elements leads to consequences and dependence on downstream elements. In this way, an effect might propagate through the system from one element to the next as a relationship. If the nature of the relationship between elements is known, then the propagation of the effect would be predictable and could be described by logic rules or other reasoning methods. One or more logic rules can then define the model of the system’s behavior.

Logic applied to a known element and its relationship to another element is the primary method to obtain a diagnosis [14-17]. An example of such a logic statement might be the conclusion that increased redness of a body part may be due to increased blood flow at a histological level, which in some cases may be an indicator of the presence of inflammatory disease. This was first stated by the Roman, Celsus, in BC 30-38 [18,19], perhaps the first recorded diagnostician.

While useful, one logic statement about one system element may not be enough to clearly conclude a specific diagnosis. Several diagnoses could still be possible with that one element’s status. For example, reddened skin could be an indication of a traumatic injury, a sunburn, a noxious chemical, or merely a person being healthy but just too hot. Fortunately, the diagnostic method can be improved by considering multiple elements together. Typically, a malfunction propagates through the system from one or a few initial elements to affect multiple other dependent elements and thus magnifying the effect. This causes a cascade of malfunctioning elements to occur together, some of which then become detectable, all together. This situation gives the opportunity to cross-reference individual logic statements at the same time. One element may logically preclude or alternatively dictate some disease diagnoses. However, multiple logic statements together can then deduce a narrower subset of diagnoses, or even a single specific diagnosis. Considering multiple elements together becomes a more powerful way of discriminating any disease present.

Rather than laboriously reasoning each logic statement with its peers, an intellectual shortcut for this technique is simply to identify the defined set of failing elements that are known to occur in a specific pattern for a specific disease. Hence, pattern recognition is the second method by which a diagnosis is made [20]. All one has to do is to remember each specific pattern that is indicative of each specific disease. An example of such pattern recognition might be used to conclude that tissue with increased redness, warmth, swelling, and pain all occurring together is suffering histological-level inflammation, such as dermatitis. These particular inflammation signs in such a pattern were, of course, first described as rubor, calor, tumor, and dolor in Roman times by Celsus [18,19]. He understood this pattern recognition.

In the example above, clearly, redness, heat, swelling, and pain are not the initial failed elements causing the inflammatory disease. The disease usually occurs as a cascade of failing elements. It is common that the initial elements which fail often are microscopic, biochemical, internal, and difficult to detect clinically. However, their failure often leads to later consequential downstream failures of external elements, which may be clinically detectable. In such cases, the hidden internal failing elements can then be logically predicted from the altered and detectable external elements in the clinical domain. We call these proxy external elements symptoms if reported by our patient, and signs if detected by the clinician, perhaps with further tests. Some elements may be both a symptom and a sign if both clinician and patient detect them. Such external signs and symptoms exist as the result of the logic rules and fall into the patterns detected by the clinical diagnostic method.

Furthermore, it can be seen that any disease process can be considered at two levels. A pathologist may describe the failing internal elements in great detail, typically on a microscopic, histological, or biochemical level [21]. The clinician meanwhile views the same disease as external failing elements, typically as clinically detectable macroscopic signs and symptoms. It is as if the one disease is being described in two languages; that of the pathologist, and that of the clinician. Clinical diagnosis translates from the language of external signs and symptoms to the corresponding internal language of pathology, which specifies the disease label. Therefore, the process of diagnosis is like a translation between these two languages. Healthcare training thus teaches the two associated sets of information for each and every disease: the internal pathological features of the medical sciences, and the corresponding clinical features of signs and symptoms. Clinicians and pathologists need to be bilingual to be able to speak both languages.

Inexperienced clinicians may need to consciously think through each logic step or pattern matching, but with training, the diagnostic process may occur without explicit thought [12,21]. Subconscious thinking has been termed System 1, as it is routine for experienced clinicians. Labored and slower conscious logical thought has been called System 2 [21,22]. It may take years of instruction and practice for a clinician to become an expert at automated System 1 correlation for many diseases. But when a diagnosis is difficult even experienced clinicians may have to resort to System 2 [21,22]. Not reverting to the slower conscious logic techniques (metacognition) where appropriate can lead to misdiagnoses [4,5,23,24], although, as expected, one report showed that very expert clinicians could diagnose so well that more time did not improve their already good accuracy [25]. Improved clinical and pathology knowledge [22] and diagnostic checklists [5,26] have also been proposed to reduce diagnostic errors.

**Figure 2:**
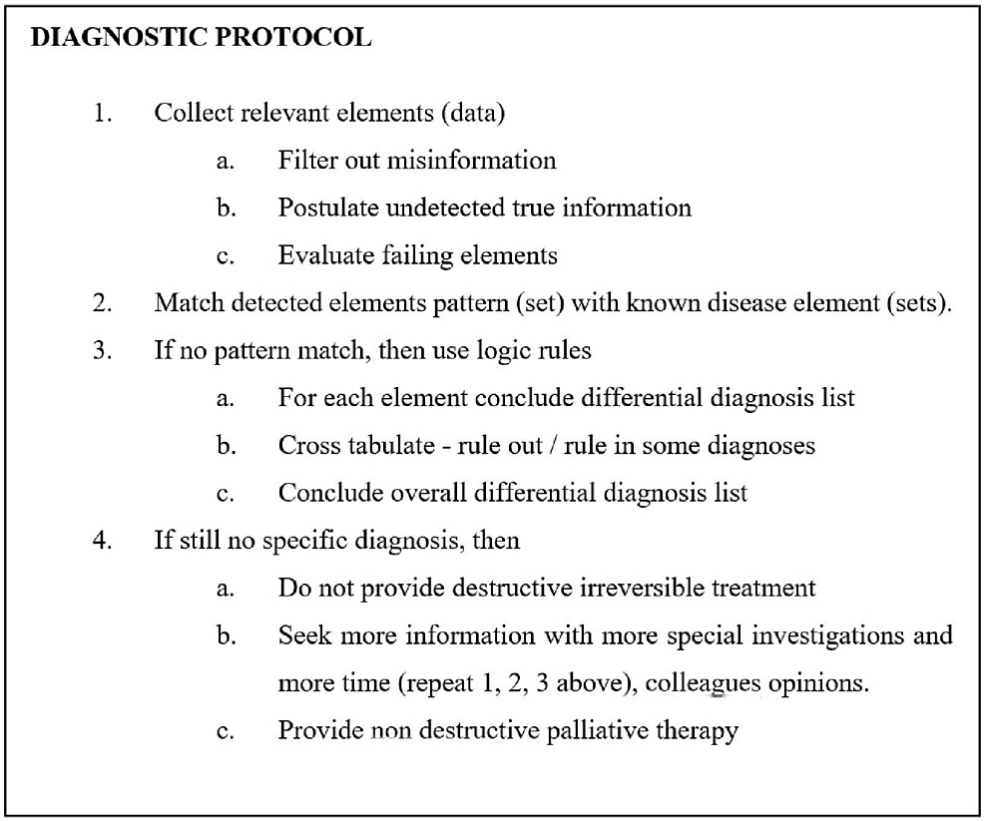
Diagnostic protocol.

How clinicians obtain and then ascribe value to clinical information has also been extensively studied [27-29]. The clinician is initially in a position of ignorance at the start of the consultation. A series of three phases then occurs: to acquire data, to form a working hypothesis of possible diagnoses, and then to test and hopefully confirm a specific diagnosis with more data [27-29]. Logic and pattern matching are used throughout, either consciously or subconsciously. Figure 2 gives a protocol of the stages of the diagnostic process. The process is iterative so that if a diagnosis is not made, then more data can be collected and the process can be repeated. Perhaps counterintuitively, more experienced clinicians were found to spend more time on the initial data acquisition than did medical students, who tended to more quickly assume a diagnosis, perhaps in error [28].

Challenges in diagnosis

Therefore, the diagnostic method seems to be relatively straightforward to do. It is just logic statements and pattern recognition of disease models. Simple, right? So, how come diagnosis can often be very difficult? This is because there are some serious practical problems that can very much hinder the process. *Imperfect System Models*

First, disease systems are usually very complicated, and not all diseases are fully described or understood. Their etiology and pathogenesis may not have been clearly established as clear logic statements or as recognizable patterns [17,30]. This ignorance can prevent diagnosis and motivates much ongoing medical research.

Undetectable Elements

**Figure 3:**
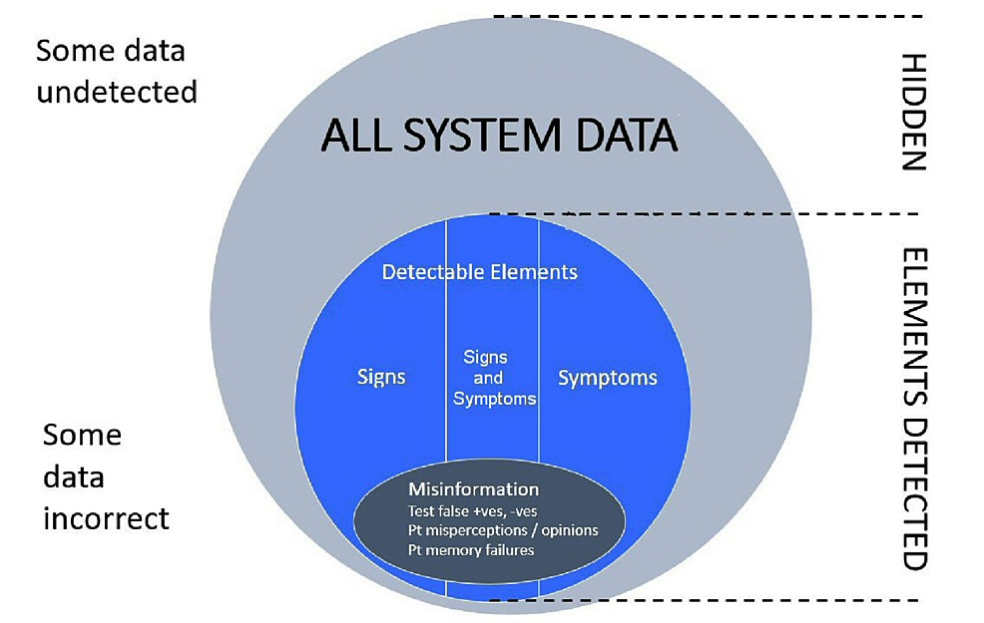
System data and its analysis for clinical diagnosis.

Second, the availability of relevant system elements is crucial to making a diagnosis. Some elements may never be detectable by any means [7]. Therefore, diagnosis relying on such totally hidden elements is impossible. This is frustrating, especially when such hidden failing elements are often causal of the disease, and so would be highly diagnostic. Figure 3 shows a Venn diagram of the relationship of the detectable information within the wider hidden information. An experienced clinician may in some cases be able to correctly interpolate absent information.

Misinformation

The clinician also may have to cope with some misinformation (Figure 3). Some of the available information may be just plain wrong. Patients forget or misconstrue the facts [31], errors occur in clinical and laboratory tests and radiographs, and mistakes may be made in recording clinical data. Good diagnostic acumen includes the ability to disregard misinformation where appropriate.

Natural Variation

The fourth practical problem is the natural variation found in all of nature. Charles Darwin explained his theory of evolution on the basis that system elements would be typically variable in a population of organisms [32]. As the environment changes, this variance permits the survival of some fortunate members of a species under new selection pressures, leading to species adaptation. While this variance is useful in species survival by evolution, it can be the bane of a clinician’s efforts to diagnose her patients. The same disease in different patients, or even in the same patient at different times, can present with a different pattern of detectable elements [17]. Some elements that are normally present may be absent. And some elements that are normally absent may be present. Indeed, there is a spectrum from health to disease, not necessarily with a clear demarcation between the two states. Significant variations in case presentations may put diagnoses in doubt. The logic and pattern recognition methods described above have to accommodate these natural variations, errors, and omissions. The diagnostic process therefore can be regarded as imperfectly defined or fuzzy in nature. Fuzzy logic [33] is required to reach a diagnosis in the presence of natural variation [17]. Rigid thinkers may be disappointed. *Workplace Limitations*

Medical workplaces are often stressful and busy environments where the workload is excessive [30,34]. Repetitive tasking and fatigue may dull the observational senses and the mind [4,5]. Moreover, some medical staff and patients may place a lower emphasis on diagnosis than on treatment when treatment may be seen as the definitive reason for the existence of the medical services. This erroneous emphasis is often reinforced by the often lesser remuneration available for diagnosis compared with treatment. All these workplace reasons may lead to greater misdiagnosis.

The limits of diagnostic method

The above problems of incomplete knowledge, hidden or misinformation, and natural variation have very real consequences. They set a limit on the diagnostic process. Clinician fatigue is also a detriment. Realistically then, in some cases, a specific diagnosis may not be possible. Often the data available may lead to only a provisional list of more likely diagnoses. This list is termed a differential diagnosis. It is essential that when a diagnosis is not certain that iatrogenically damaging treatments are not carried out. In all but emergency situations, it is better to seek more information with further testing, with further opinions from more experienced colleagues, or with more time, such that the true state of the patient can become apparent [35,36].

Diagnosis and education

It can be seen from the above analysis that like many other skills, initially, the diagnosis has to be consciously learned [12]. At first, we may not realize the full extent of our lack of skill. This has been termed unconscious incompetence [37]. With education, a person moves on to realize they are unskilled (conscious incompetence), then hopefully with much intellectual analysis and effort they become consciously competent (System 2 behavior [21]). Finally, when they are very experienced they may become unconsciously competent (System 1 behavior [21]) and be able to perform the task without specifically analyzing each step. This learning transition was first proposed by Noel Burch in the 1970s for training staff in businesses and is now an established educational model [37,38]. The final unconscious competence relies largely on what might be described as intuition, learned by years of experience [30,39]. But in the earlier learning stages, the clinician must use rigorous conscious logic and pattern recognition to laboriously conclude the diagnosis [12]. Hence, an emphasis by educators should be to provide clear and detailed explanations of the known logic rules and the patterns of both pathological and clinical elements associated with each specific state of health and disease. The student will then have some rational framework with which to consciously translate the external signs and symptoms into internal diagnoses of pathosis [40]. Experienced clinical educators who can not deconstruct their own unconscious competence into defined conscious and rational statements will not optimize their students’ learning. The art of diagnosis needs to be transformed into an explicit and explainable science to be readily teachable to inexperienced operators [12].

Clinical diagnostic adages

No account of the clinical diagnostic process would be complete without mentioning some clinical adages that are in common use.

First, it has been said that “one’s ears are the best diagnostic tool,” with evidence from a patient’s history often trumping complex and expensive special investigations [35]. More emphasis on the clinical history and examination is helpful before special investigations are requested.

Second, any reported symptoms should be confirmed with a related clinical sign. Reproducing the symptom with an associated clinical sign confirms the validity of the history.

Third, another useful concept is, where appropriate, to try to deduce an individual diagnosis for each tissue, process, or body part under investigation. This more detailed diagnosis by individual sites can lead to a more precisely tailored treatment plan. Each of these tissues or body parts may need specific but different management at different time stages.

Fourth, the adage that “common things are common” reminds us of the greater probability of a routine condition compared with an extraordinarily rare disease, notwithstanding the need to discount rare conditions.

Finally, when a diagnosis is still in doubt, in all but the most extreme circumstances, it is critical that harmful intervention is not provided until a dependable diagnosis is obtained. Repetition of the diagnostic process, additional investigations, further opinions from colleagues, or just allowing more time may lead to a clear diagnosis. *Primum non nocere*.

## Conclusions

In summary, misdiagnoses in healthcare are common and cause much harm and cost. The diagnostic method uses two fundamental reasoning techniques: individual or multiple logic statements, and pattern recognition of failing disease elements. Many elements are out of view of the clinician who must reverse translate the external signs and symptoms into internal diseased elements to deduce the internal diagnosis. While experienced clinicians may be able to do much of this reasoning subconsciously, inexperienced clinicians must consciously deliberate each logic step and pattern. Although observation of clinical cases is a valuable learning exercise, clinical teachers will accelerate their students’ learning by providing the deconstructed reasoning behind each diagnosis. The aim here is to better rationalize the diagnostic process, to allow it to be better taught, and to allow clinicians to become better diagnosticians. Without knowing disease from health, or one disease from another, reliable design and delivery of therapy are not possible. It is sobering to realize that someone over 300 years ago knew this when they coined the word “diagnosis” to “know apart.”

## References

[1] Singh H, Schiff GD, Graber ML, Onakpoya I, Thompson MJ (2017). The global burden of diagnostic errors in primary care. BMJ Qual Saf.

[2] Kohn L, Corrigan J, Donaldson MS (2000). To Err is Human: Building a Safer Health System. https://www.nap.edu/download/9728.

[3] Graber M, Gordon R, Franklin N (2002). Reducing diagnostic errors in medicine: what's the goal?. Acad Med.

[4] Croskerry P (2003). The importance of cognitive errors in diagnosis and strategies to minimize them. Acad Med.

[5] Ely JW, Graber ML, Croskerry P (2011). Checklists to reduce diagnostic errors. Acad Med.

[6] Newton CW, Zunt SL (1987). Endodontic intervention in the traumatic bone cyst. J Endod.

[7] Newton CW, Hoen MM, Goodis HE, Johnson BR, McClanahan SB (2009). Identify and determine the metrics, hierarchy, and predictive value of all the parameters and/or methods used during endodontic diagnosis. J Endod.

[8] Makary MA, Daniel M (2016). Medical error-the third leading cause of death in the US. BMJ.

[9] Singh H, Meyer AN, Thomas EJ (2014). The frequency of diagnostic errors in outpatient care: estimations from three large observational studies involving US adult populations. BMJ Qual Saf.

[10] Lipczak H, Dørflinger LH, Enevoldsen C, Vinter MM, Knudsen JL (2015). Cancer patients’ experiences of error and consequences during diagnosis and treatment. Patient Exp J.

[11] Young M, Thomas A, Lubarsky S (2018). Drawing boundaries: the difficulty in defining clinical reasoning. Acad Med.

[12] Nkanginieme KE (1997). Clinical diagnosis as a dynamic cognitive process: application of Bloom’s taxonomy for educational objectives in the cognitive domain. Med Educ Online.

[13] (2021). Google search “Diagnosis Etymology.” Online etymology dictionary. https://www.google.com.au/search.

[14] Reiter R (1987). A theory of diagnosis from first principles. Artif Intell.

[15] Ledley RS, Lusted LB (1959). Reasoning foundations of medical diagnosis; symbolic logic, probability, and value theory aid our understanding of how physicians reason. Science.

[16] Popper KR (1959). The Logic of Scientific Discovery.

[17] Seising R (2006). From vagueness in medical thought to the foundations of fuzzy reasoning in medical diagnosis. Artif Intell Med.

[18] Woolf N (2000). Cell, Tissue and Disease: The Basis of Pathology.

[19] Punchard NA, Whelan CJ, Adcock I (2004). The Journal of Inflammation. J Inflamm (Lond).

[20] Bolon-Canedo V, Remeseiro B, Alonso-Betanzos A (2016). Machine learning for medical applications. ESANN 2016 proceedings, European Symposium on Artificial Neural Networks, Computational Intelligence and Machine Learning.

[21] ten Cate O, Durning SJ (2018). Approaches to assessing the clinical reasoning of preclinical students. Principles and Practice of Case-Based Clinical Reasoning Education.

[22] Norman GR, Monteiro SD, Sherbino J, Ilgen JS, Schmidt HG, Mamede S (2017). The causes of errors in clinical reasoning: cognitive biases, knowledge deficits, and dual process thinking. Acad Med.

[23] Graber M (2003). Metacognitive training to reduce diagnostic errors: ready for prime time?. Acad Med.

[24] Coderre S, Wright B, McLaughlin K (2010). To think is good: querying an initial hypothesis reduces diagnostic error in medical students. Acad Med.

[25] Sherbino J, Dore KL, Wood TJ, Young ME, Gaissmaier W, Kreuger S, Norman GR (2012). The relationship between response time and diagnostic accuracy. Acad Med.

[26] Winters BD, Aswani MS, Pronovost PJ (2011). Commentary: reducing diagnostic errors: another role for checklists?. Acad Med.

[27] Gale J (1980). The Diagnostic Thinking Process in Medical Education and Clinical Practice. https://discovery.ucl.ac.uk/id/eprint/10019046.

[28] Gale J, Marsden P (1982). Clinical problem solving: the beginning of the process. Med Educ.

[29] Maupomé G, Schrader S, Mannan S, Garetto L, Eggertsson H (2010). Diagnostic thinking and information used in clinical decision-making: a qualitative study of expert and student dental clinicians. BMC Oral Health.

[30] Jones R (1991). Treatment outcome as a diagnostic tool. Mod Med (South Africa).

[31] Merckelbach H, Jelicic M, Pieters M (2011). Misinformation increases symptom reporting: a test - retest study. JRSM Short Rep.

[32] Rasmussen C, Bisanz J (2009). Executive functioning in children with fetal alcohol spectrum disorders: profiles and age-related differences. Child Neuropsychol.

[33] Zadeh L (1965). Fuzzy sets. Inf Control.

[34] Amin MM, Graber M, Ahmad K (2012). The effects of a mid-day nap on the neurocognitive performance of first-year medical residents: a controlled interventional pilot study. Acad Med.

[35] Balogh EP, Miller BT, Ball JR (2015). Improving Diagnosis in Health Care. https://www.ncbi.nlm.nih.gov/books/NBK338596/.

[36] Zwaan L, Singh H (2015). The challenges in defining and measuring diagnostic error. Diagnosis (Berl).

[37] Catlin D, Csizmadia A, Omeara J, Younie S (2015). Using educational robotics research to transform the classroom. 6th International Conference on Robotics in Education.

[38] Gordon Training International (2021). Learning a new skill is easier said than done. https://www.gordontraining.com/free-workplace-articles/learning-a-new-skill-is-easier-said-than-done/.

[39] Davies P (1987). Diagnosis: the need for demystification. Lancet.

[40] Olson AP, Graber ML (2020). Improving diagnosis through education. Acad Med.

